# Chitosan-Coated Poly(lactic acid) Nanofibres Loaded with Essential Oils for Wound Healing

**DOI:** 10.3390/polym13162582

**Published:** 2021-08-04

**Authors:** Giulia Milanesi, Barbara Vigani, Silvia Rossi, Giuseppina Sandri, Elisa Mele

**Affiliations:** 1 Department of Drug Sciences, University of Pavia, V.le Taramelli 12, 27100 Pavia, Italy; giulia.milanesi02@universitadipavia.it (G.M.); barbara.vigani@unipv.it (B.V.); silvia.rossi@unipv.it (S.R.); g.sandri@unipv.it (G.S.); 2 Materials Department, Loughborough University, Loughborough LE11 3TU, UK

**Keywords:** electrospinning, chitosan, essential oils

## Abstract

Chronic skin wounds are characterised by a non-healing process that makes necessary the application of wound dressings on the damaged area to promote and facilitate the recovery of skin’s physiological integrity. The aim of the present work is to develop a bioactive dressing that, once applied on the injured tissue, would exert antibacterial activity and promote adhesion and proliferation of fibroblasts. Nanofibres consisting of poly(lactic acid) (PLA) and essential oils (EOs) were electrospun and coated with a medium molecular weight chitosan (CS). Black pepper essential oil (BP-EO) or limonene (L), well-known for their antibacterial properties, were added to the PLA/acetone solution before electrospinning; phase separation phenomena occurred due to the poor solubility of the EOs in the PLA solution and led to fibres having surface nano-pores. The porous electrospun fibres were coated with CS to produce hydrophilic membranes that were easy to handle, biocompatible, and suited to promote cellular proliferation. The fibrous scaffolds were tested in terms of mechanical resistance, wettability, antibacterial activity, in-vitro cytotoxicity, and ability to promote fibroblasts’ adhesion and proliferation. The results obtained proved that the CS coating improved the hydrophilicity of the fibrous mats, enhanced EO’s antibacterial potential, and promoted cell adhesion and proliferation.

## 1. Introduction

Wound healing is a dynamic physiological process, process that involves a multitude of cellular, humoral, and molecular events, with the aim of restoring the integrity and functionality of injured skin [1,2]. The breakdown of such complex pathway, as a consequence of repeated tissue insults, persistent bacterial infections and underlying pathologies, could result in a delayed or impaired healing process, from which chronic wounds arise [3,4]. Nowadays, chronic wounds are the major cause of morbidity worldwide and the healthcare costs related to wound care are a billion-dollar issue [5]. In this scenario, there is an increasing interest in the development of bioactive wound dressings that are functional to limit bacterial colonization of the wound bed and, thus, prevent infections and sepsis [6,7,8,9,10].

Electrospinning is a highly versatile technique used in the tissue engineering field, which enables the production of solid fibrous membranes by applying an electric field to polymer-based solutions [11,12]. Membranes made of electrospun nanofibres are considered innovative wound dressings as they physically protect the wound bed, mimic the native structure of the extracellular matrix (ECM), adsorb wound exudates and prevent wound contamination, while supporting cell migration, adhesion and proliferation as well as angiogenesis [13,14,15,16,17]. The high surface to volume ratio of electrospun nanofibres promotes the release of loaded drugs and/or biomolecules and maximizes the intimate contact with the cells of the wound environment, accelerating tissue regeneration mechanisms [18,19]. Among the various topographical cues provided by such nanosystems, surface roughness and porosity play a pivotal role in favouring cell attachment and proliferation [17,20]. The solvents used for the preparation of polymer-based solutions to be electrospun are crucial in the production of fibres with a porous surface: it has been demonstrated that solvent mixtures characterized by different evaporation rates allow the formation of fibres with surface pores [21]. Several studies in the literature have shown that fibrous scaffolds with a high surface porosity can be prepared by electrospinning polymer/solvent/non-solvent ternary systems, thus by non-solvent induced phase separation [22,23,24,25].

Essential oils (EOs) are complex mixtures of various volatile compounds, mainly terpenoids and phenylpropanoids, which are typically extracted from the non-woody part of aromatic plants [26,27,28]. EOs exhibit a wide range of bioactive properties, such as antioxidant, antiviral, anticancer, insecticidal, anti-inflammatory and anti-allergic, but, in the literature, they are primarily known for their antimicrobial activity [29,30,31,32]. Indeed, thanks to their hydrophobic nature, EOs can link phospholipids and lipids constituting the bacteria cell membrane with a consequent alteration of its permeability to ions and cytoplasmic components that could result in cell lysis [33,34]. Therefore, in an attempt to produce promising bioactive dressings for wound healing, the incorporation of EOs, such as cinnamon [35], lemongrass, peppermint [34], tea tree, thyme [36], lavender [37], clary sage and black pepper [25], in electrospun fibres has been proposed.

In a previous work of ours, it has been demonstrated that the addition of EOs to a poly(lactic acid) (PLA) solution to be electrospun played a pivotal role in controlling fibre surface topography [25]. In particular, the inclusion of EOs that were poorly miscible with PLA and rich in volatile components led to thermodynamic instabilities and phase separation events during the electrospinning process. This resulted in fibres with a nano-textured surface.

Given this premise, the present work deals with the development of a multifunctional wound dressing, easy to handle and apply to the injured area, endowed with antibacterial properties and able to enhance cell adhesion and proliferation; such a dressing should prevent wound chronicization and accelerate tissue repair mechanisms. In particular, nano-textured fibres, composed of PLA and EOs, were prepared through the electrospinning technique and, subsequently, coated with chitosan (CS). Black pepper essential oil (BP-EO) and limonene (L), one of the main BP components, were selected as bioactive agents for both their well-known antimicrobial activity and capability to control fibre surface topography. The CS coating created a hydrophilic layer onto the fibre surface that enhanced cell adhesion and has potential to promote tissue repair and regeneration [2,10].

CS is a cationic polysaccharide that represents one of the most attractive biomaterials for wound healing applications due to its biocompatibility, biodegradability, free-radical scavenging, haemostatic, mucoadhesive and antibacterial properties [38]. Owing to its unique characteristics, CS properties are relevant to all stages of wound healing, since CS acts as a chemo-attractant for neutrophils and macrophages, inhibits matrix metalloproteinases and provides an anti-infective effect against a variety of pathogens [2]. Concerning this latter property, it has been demonstrated that CS cationic amino groups interact with the anionic ones on the bacterial cell wall, affecting its permeability and suppressing the biosynthesis of the wall itself. Other mechanisms proposed in the literature to explain CS antimicrobial activity contemplate the inhibition of cell exchanges and nutrient absorption, due to the generation of a polymeric shell around bacteria, and the chelation of metals, which are pivotal for microbial growth [39,40].

In this work, electrospun PLA fibres, containing BP-EO and L, were characterized in terms of morphological and mechanical properties, before and after coating with CS. In order to confirm the presence of a CS layer, that improves the hydrophilicity of the fibrous membranes, the wettability of the electrospun fibres was evaluated through water contact angle measurements (before and after CS coating). Fibrous membrane effectiveness against different bacteria strains was assessed, as well as their biocompatibility and capability to promote fibroblast adhesion and proliferation.

## 2. Materials and Methods

### 2.1. Chemicals

Poly(lactic acid) (PLA 4060D, MW = 120,000 g/mol, amorphous polymer with an L-lactide content of around 88 wt%; NatureWorks LLC, Minnetonka, MN, USA), black pepper essential oil (BP-EO, extracts of *Piper nigrum*; Freshskin Beauty Ltd, Nottingham, UK), limonene (L, C_10_H_16_ MW = 136 g/mol; Sigma Aldrich, Gillingham, UK) and acetone (Sigma Aldrich, Gillingham, UK) were used for the preparation of electrospun fibres. Medium molecular weight chitosan (CS; Sigma Aldrich, Gillingham, UK), glacial acetic acid (Sigma Aldrich, Gillingham, UK), Triton X-100 (TX-100; Fluka BioChemika, Buchs, St. Gallen, Switzerland) and deionized water were used for the preparation of the coating solution.

For experiments with Normal Human Dermal Fibroblasts (NHDF) from juvenile foreskin (PromoCell GmbH, VWR, Milan, Italy), the materials hereafter reported were used: dimethyl sulfoxide (DMSO), Dulbecco’s Modified Eagles Medium (DMEM), Dulbecco’s Phosphate Buffer Solution (PBS), inactivated foetal bovine serum (FBS), MTT (3-(4,5-dimethylthiazol-2-yl)-2,5-diphenyltetrazolium bromide), antibiotic/antimycotic Solution (100×; stabilized with 10,000 units penicillin, 10 mg streptomycin and 25 μg amphotericin B per mL), trypan blue solution and trypsin–EDTA solution were purchased from Sigma-Aldrich (Milan, I). Glutaraldehyde solution (Sigma-Aldrich, Milan, I) was used to fix the cells on fibres prior to scanning electron microscopy (SEM) and confocal-laser scanning microscopy (CLSM) analyses. Phalloidin-TRITC (Tetramethylrhodamine B isothiocyanate) and Hoechst 33258, purchased from Sigma Aldrich (Milan, Italy), were used for the staining of cell cytoskeleton and nuclei, respectively.

### 2.2. Preparation of PLA-Based Solutions

PLA solutions were prepared by dissolving the polymer in acetone at the concentration of 14% *w*/*v*, under magnetic stirring overnight until complete dissolution [25]. PLA solutions containing black pepper essential oil (PLA-BP) and limonene (PLA-L) were prepared by dissolving 14% *w*/*v* PLA in acetone and then adding 10% *v*/*v* of BP-EO or L. PLA-BP and PLA-L solutions were maintained under magnetic stirring overnight until complete dissolution. PLA is soluble in acetone, whereas it has poor solubility in both BP-EO and limonene.

### 2.3. Preparation of PLA-Based Fibres by Electrospinning

Electrospun PLA-based fibres (PLA, PLA-BP, and PLA-limonene) were produced using a horizontal electrospinning apparatus (S1500032-0, Linari Engineering s.r.l., Pisa, Italy). The equipment consisted of a high voltage power supply, a syringe pump (New Era Pump System, NE-300, Farmingdale, NY, USA) with a plastic syringe (23G needle) containing the polymer solution, two electrodes, a plexiglass collector to which a metallic support was fixed. In order to obtain a continuous jet that could originate fibres without defects, the operating conditions of spinning were set as follows: flow rate of 0.7 mL/h, voltage of 10 kV and collector-needle distance of 15 cm. All experiments were conducted in normal environmental conditions.

### 2.4. CS Coating of PLA-Based Fibres

The coating solution was prepared at room temperature by dissolving 0.5% *w*/*w* CS and 0.2% *w*/*w* TX-100 in an aqueous solution of 1% *v*/*v* acetic acid and left under magnetic stirring overnight. Electrospun PLA-based fibres were soaked in the coating solution for 3 h, washed in deionized water and then dried at room temperature. The CS coated PLA-based fibres were named cPLA, cPLA-BP (containing BP EO) and c-PLA-L (containing limonene).

### 2.5. Characterisation of PLA-Based Fibres

The morphological properties of PLA-based fibres, before and after CS coating, were investigated by SEM. The analyses were performed using two different instruments: ZEISS Gemini SEM 450 microscope (Carl Zeiss, Ostalbkreis, Germany) and JSM-7800F Schottky Field (Jeol, MA, USA). Samples were made conductive by vacuum-phase vapor deposition by using Quorum Q150R-S sputter (Emitech SC7640 Sputter Coater, Polaron, Laughton, UK) with a current of 20 mA, time 90 s and material Au/Pd (80:20). A second set of images were obtained using Mira3 XMU (Tescan, Brno-Kohoutovice, Czech Republic). The samples were rendered conductive by vacuum-phase platinum deposition for 60 s. Electrospun fibre dimensions were measured using the imaging analysis program ImageJ 2.0 (net.imagej: imagej:2.0.0-rc-55, Java-based operating system, 2009); thirty fibres were considered for each sample.

Ethanol was used to extract BP-EO and limonene from the PLA-based fibres (PLA-BP, PLA-L, cPLA-BP and cPLA-L). Fibre mats (0.02 g) were submerged in ethanol (10 mL) and stirred for 24 h. The amount of BP-EO and limonene was estimated using a UV-Visible spectrophotometer (UV-Vis-NIR Cary 5000, Agilent) at a wavelength of 264 nm for BP-EO and 234 nm for limonene, based on calibrations curves. The encapsulation efficacy (or entrapment efficiency, EE) and loading capacity (LC) were calculated as [41]:

EE (%) = (*V_e_*/*V_t_*) × 100 (1)

LC (%) = (*m_e_*/*m_t_*) × 100 (2)

where *V_e_* is the amount (mL) of BP-EO or limonene released by the fibres in ethanol (estimated using calibration curves); *V_t_* is the amount (mL) of BP-EO or limonene mixed in the PLA solution prior to electrospinning; *m_e_* is the amount (mg) of BP-EO or limonene released by the fibres in ethanol (estimated using calibration curves); *m_t_* is mass (mg) of electrospun fibres used for the extraction.

The glass transition temperature (*T_g_*) of the electrospun mats was investigated by differential scanning calorimetry (DSC, TA Instruments Calorimetric Analyser, Elstree, UK) in the temperature range between −20 °C and 100 °C. The tests were performed at a heating/cooling rate of 10 °C/min in three scans. The onset point of the temperature step change of each curve was used as glass transition temperature (second heating run).

The mechanical properties of the PLA-based fibres, before and after CS coating, were investigated in tension using the TA.XT plus Texture Analyzer (Stable Micro Systems, Godalming, UK), equipped with 5 kg load cells and an A/TG tensile grips probe. Each sample was cut (1 cm × 3 cm) and inserted between the two clamps so that the gauge length was 1 cm. The analyses were performed by setting a trigger force of 1 g, a constant rising speed of 5 mm/s and a rising distance of 15 mm. Tensile strength, elongation at break and toughness (area under the stress-strain curve) were calculated for both uncoated and coated fibres. Each test was run in triplicates.

Before and after CS coating, fibre wettability was evaluated through water contact angle measurements. The equipment (DataPhysics OCA 20, Filderstadt, Germany) consists of a horizontal plane on which the sample is placed. Above the plane, a vertical syringe, equipped with a thin needle able to deposit an exact amount of deionized water (5 μL), is positioned. Once the drop is released, a recording time of 5 min is set; instant images of the drop are automatically saved from the video every 5 s, starting from time 0 (T0) for the entire recording time. The contact angle of the uncoated fibres (PLA, PLA-BP, and PLA-L) was calculated at T0 and after 5 min (T5 min) since they were expected to be hydrophobic. The contact angle of CS coated fibres (cPLA, cPLA-BP, and cPLA-L) was evaluated at T0 and after 10 s (T10 s). Each test was run in triplicates.

### 2.6. Antibacterial Tests

The antimicrobial activity of the fibres, before and after CS coating, was evaluated against different bacteria strains: *Staphylococcus aureus* ATCC 6538, *StaFiylococcus epidermidis* ATCC 12228, *Escherichia coli* ATCC 10356, and *Pseudomonas aeruginosa* ATCC 15442. In particular, the killing time was determined as the exposure time required to kill a standardized microbial inoculum [42]. Before testing, the bacteria were grown in Tryptone Soya Broth (Oxoid; Basingstoke, UK) at 37 °C overnight. Subsequently, they were centrifuged at 2000 rpm for 20 min to separate cells from broth and then suspended in PBS (pH = 7.3). The suspension was diluted to adjust the number of cells to 1 × 10^7^–1 × 10^8^ CFU/mL (colony-forming units/mL).

Pieces of fibrous membranes (uncoated and CS coated) were added to the microbial suspensions at the concentration of 2.5 mg/mL and then incubated at 37 °C. For each bacteria strain, a suspension without sample was prepared in PBS as control. Viable cell count was performed on each microbial suspension after 0 and 24 h of contact with each sample and on the relevant control; bacterial colonies were enumerated in Tryptone Soya Agar (Oxoid; Basingstoke, UK) after incubation at 37 °C for 24 h. For each bacteria strain and contact time, the microbicidal effect value (ME) of each sample was calculated according to the following equation [43]:

*ME=log(Nc)−log (Nd)* (3)

where *Nc* indicates the number of CFU of the control microbial suspension and *Nd* represents the number of CFU of the microbial suspension containing the fibrous membrane. Each test was run in triplicates.

### 2.7. In-Vitro Cell Culture Tests

Normal human dermal fibroblasts (NHDF) from 6th to 12th passage were used. Cells were cultured in complete culture medium (CM) in the presence of DMEM, supplemented with 10% FBS and 1% *v*/*v* antibiotic/antimycotic solution. After 24 h UV-irradiation, uncoated and CS-coated PLA-based fibres were cut into pieces having a surface area of approximately 0.32 cm^2^, placed in a 96-well plate and seeded with NHDF cells at a density of 10,000 cells/cm^2^. Thereafter, cells were maintained in incubator (Shellab^®^ Sheldon^®^ Manufacturing Inc., Cornelius, OR, USA) at 37 °C in a 5% CO_2_ atmosphere with 95% relative humidity.

The effect of PLA-based fibres on cell viability was evaluated over 3 days by a MTT assay, using CM as control. Briefly, culture medium was removed, and cells were washed using PBS; subsequently, 50 μL of MTT 7.5 μM in 100 μL of DMEM without phenol red were added to each well and incubated for 3 h (37 °C and 5% CO_2_). After this time, MTT solution was removed from each well and 100 μL of DMSO, used as solubilising agent, were added. In order to promote the complete dissolution of formazan crystals, obtained from MTT dye reduction by mitochondrial dehydrogenases of live cells, the solution absorbance was measured by means of an iMark® Microplate reader (Bio-Rad Laboratories S.r.l., Segrate, Italy) at a wavelength of 570 nm and 690 nm (reference wavelength) after 60 s of mild shaking. Results were expressed as cell viability (% CM) by normalising the absorbance measured after contact with each sample with that measured for CM. Five replicates were performed for each sample.

A morphological study of fibroblasts on uncoated and CS-coated fibres was carried out after 7 days of culture. Cell distribution within each fibrous membrane was appreciated using a SEM Mira3 XMU (Tescan, Brno-Kohoutovice, CZ). After medium removing, the cell-fibre systems were rinsed with PBS and fixed in 3% *v*/*v* glutaraldehyde solution in PBS for at least 3 h. Samples were further rinsed with PBS, dehydrated by means of ethanol solutions at increasing concentrations (25%, 50%, 75% and 100% *v*/*v*) and dried at room temperature in a clean bench. The morphological analysis of the coated fibres was carried out by SEM, as previously described.

Cell distribution within each fibrous membrane was appreciated by means of confocal laser scanning microscopy (CLSM). After medium removing, the cell-fibre systems were rinsed with PBS and then fixed in 3% *v*/*v* glutaraldehyde solution in PBS for at least 3 h. Afterwards, the cells were permeabilized in 0.1% *v*/*v* Triton X-100 in PBS for 5 min and, then, cellular cytoskeletons were stained by incubating with 50 µL (50 µg/mL) Phalloidin-TRITC (Tetramethylrhodamine B isothiocyanate) for 45 min, in the dark. Then each cell-fibre system was washed twice with PBS and cell nuclei were stained with 100 µL of Hoechst 33258 diluted 1:10,000 for 15 min in the dark. Cell-fibre systems were placed on a microscope slide and imaged using a CLSM (Leica TCS SP2, Leica Microsystems, Milan, Italy) with λex = 346 nm and λem = 460 nm for Hoechst 33258 and λex = 540 nm and λem = 570 nm for Phalloidin-TRITC. The acquired images were processed with software associated with the microscope (Leica Microsystem, Milan, Italy).

### 2.8. Statistical Analysis

Whenever possible, experimental values of the various types of measurements were subjected to statistical analysis carried out using the statistical package Statgraphics 5.0 (Statistical Graphics Corporation, The Plains, VA, USA). One-way ANOVA-Multiple Range Test was used.

## 3. Results and Discussion

Poly(lactic acid) can be electrospun from solutions having a concentration between 12% and 15% *w*/*v* [24,44,45]. In the last years, essential oils have been encapsulated in PLA electrospun fibres for wound care and food packaging applications [24,25]. Liu and co-workers prepared novel antimicrobial dressings by incorporating cinnamaldehyde (CA)/β-cyclodextrin (β-CD) in PLA nanofibres, which has been shown to guarantee a long-lasting antibacterial effect against both *Escherichia coli* and *Staphylococcus aureus*, without exerting any cytotoxic effects on human skin fibroblasts [21]. Zhang et al. investigated the effect of tea tree and manuka EOs on the mechanical properties and antibacterial activity of electrospun PLA fibres [24]. EOs reduced the fibre glass transition temperature up to 60% and increased their elongation-at-break and tensile strength up to 12 times, acting as plasticisers. Moreover, manuka EO was able to block the formation of biofilms of *Staphylococcus epidermidis*.

**Figure 1:**
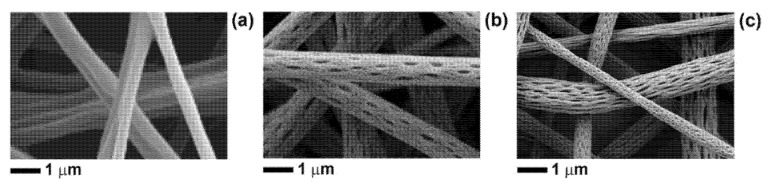
Selection of SEM micrographs of (**a**) PLA fibres, (**b**) PLA-BP fibres, and (**c**) PLA-limonene fibres to show the surface features.

In one of our previous works, it has been proven that the addition of black pepper (BP) EO to a 14% *w*/*v* PLA-acetone solution induced a phase separation phenomenon and, thus, thermodynamic instabilities, which led to the formation of fibres with a nano-porous surface [25]. The phase-separation events were attributed to the fast evaporation of the most volatile components of BP-EO, in combination with their poor miscibility with PLA (while acetone is considered a solvent for PLA). It was observed that the nano-pore density on the surface of the fibres was strictly related to the amount of EO used; in particular, a BP-EO concentration equal to 10% *v*/*v* was suitable to obtain a uniform distribution of well-defined pores along the main fibre axis (that was the direction of polymer stretching during the electrospinning). Tests were also conducted to assess the effect of limonene, that is one of the main highly volatile components of BP-EO, on the morphology of the PLA electrospun fibres. Since PLA is insoluble in limonene, the ternary blend PLA/acetone/limonene generated fibres with a surface porosity more evident than that one obtained using BP-EO. In the present work, 14% *w*/*v* PLA-acetone solutions, containing either BP-EO or limonene, at a concentration of 10% *v*/*v*, were electrospun to produce PLA-BP and PLA-limonene nano-textured fibres. In Figure 1, SEM images of the electrospun fibres are shown.

All types of fibres are free from defects, and those containing either BP-EO or limonene are characterised by well-defined and elongated surface pores, in agreement with the previous study [25]. As discussed before, the morphology of both PLA-BP fibres and PLA-limonene fibres is determined by phase separation events happening during the electrospinning process, when polymer-rich (mainly PLA in acetone) and polymer-lean domains (mainly BP or limonene) form due to the limited solubility of PLA in essential oils (BP-EO or limonene) and the rapid evaporation of solvent/non-solvent. The evaporation of the non-solvents from the polymer-lean regions leads to surface pores, while the applied electric force (during electrospinning) causes the pores to stretch. As shown in Figure 1b,c, the pores are elongated along the main axis of the fibres, which is the direction of polymer stretching during electrospinning. The number of pores per unit surface area is lower for PLA-BP fibres than for PLA-limonene fibres, because BP-EO contains limonene but also other chemical compounds that are less volatile than limonene [25].

**Figure 2:**
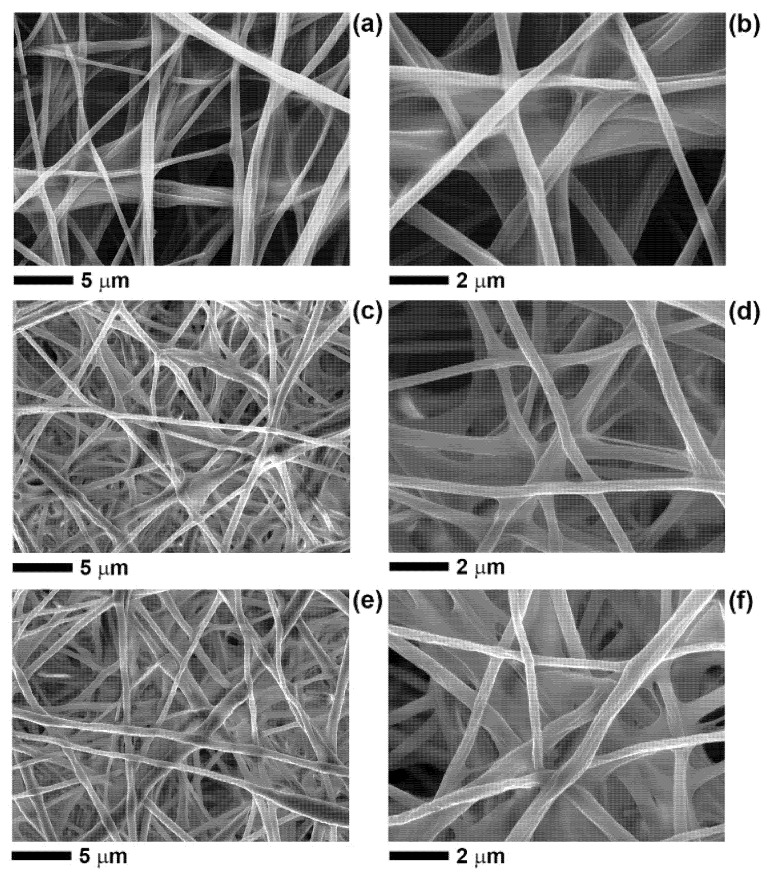
SEM micrographs, at different magnification, of CS-coated fibres: (**a**,**b**) cPLA, (**c**,**d**) cPLA-BP, (**e**,**f**) cPLA-limonene.

The hydrophilicity and bioactivity of the PLA-based fibres, containing BP-EO and limonene, were enhanced by coating the fibres with chitosan (CS), a cationic polysaccharide well known as wound healing enhancer [2,10,38]. The solution used (acidic-aqueous solution containing 0.5% *w*/*w* CS and 0.2% *w*/*w* TX-100) produced a homogeneous coating onto the fibres’ surface, as shown in Figure 2. The addition of a surfactant, such as TX-100, was necessary to reduce the interfacial tension of the coating solution and enable the complete wetting of the hydrophobic fibrous membranes. The CS solution wetted the whole volume of the electrospun mats and, after solidification, a thin polymeric film formed around the fibres and, in some regions, it was visible in between fibres (Figure 2).

The CS coating was present not only on the outer surface of the electrospun membranes but also on the inner layers. The effect of the CS treatment was particularly evident for cPLA-BP and cPLA-limonene fibres, whose surface nanopores were filled with CS and therefore disappeared. Nevertheless, the concentration of CS used preserved the overall fibrous structure without clogging the pores in between fibres.

**Figure 3:**
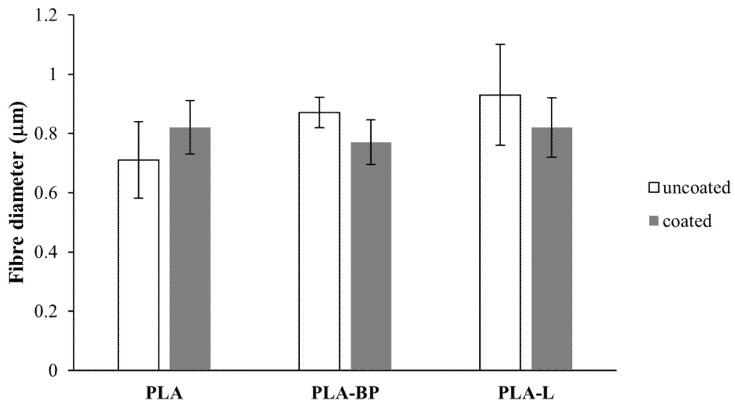
Mean diameter of PLA, PLA-BP and PLA-limonene (PLA-L) fibres, before and after CS coating (mean values ± s.d.; n = 30); one-way ANOVA (*p* < 0.05), PLA vs. PLA-L; PLA-BP vs. PLA-L.

The mean diameter of the PLA-based fibres is reported in Figure 3. No statistically significant differences can be observed in the fibres’ diameter before and after CS treatment, indicating that the CS coating did not alter the size of the fibres.

The amount of BP-EO and limonene contained within the PLA fibres (uncoated and CS-coated) was quantified by UV-visible spectroscopy. The encapsulation efficiency of PLA-BP fibres and cPLA-BP fibres was around 65% and 56%, respectively, corresponding to a loading content of ~25% (for PLA-BP) and ~21% (for cPLA-BP). It is expected that the chitosan coating limited the extraction of BP and limonene, hence determining a lower encapsulation efficiency for the chitosan-coated fibres. A lower entrapment efficiency was calculated for the PLA-limonene fibres (~43%, corresponding to a loading content of ~16%), possibly due to the higher evaporation rate of limonene during electrospinning with respect to BP-EO [25]. In the literature, encapsulation efficiency in the range of 8% 67% has been reported for poly(vinyl alcohol) fibres containing limonene, depending on environmental parameters during fibre formation, such as relative humidity and temperature [46,47].

The effect of BP-EO and limonene on the glass transition temperature (*T_g_*) of the electrospun mats was investigated by DSC. Values of *T_g_* of (52 ± 2) °C were measured for PLA fibres, while the addition of BP-EO and limonene determined a decrease of *T_g_* to (32 ± 2) °C and (20 ± 2) °C, respectively, for both uncoated and CS-coated mats. This is in agreement with previous research where essential oils have been reported to work as plasticisers for PLA [24,25].

**Figure 4:**
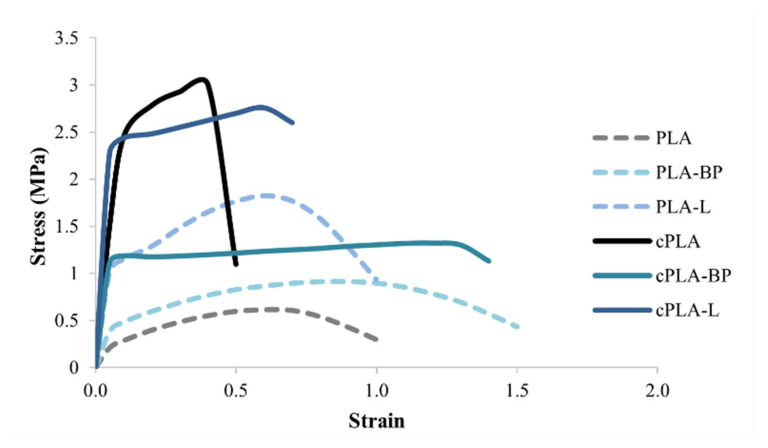
Average stress-strain curves of uncoated (PLA, PLA-BP and PLA-L) and coated (cPLA, cPLA-BP and cPLA-L) fibres (mean value ± s.e.; n = 3).

The mechanical properties of the fibres, before and after CS coating, were investigated by tensile tests; in particular, stress-strain curves were used to evaluate the tensile strength and the elongation at break of both uncoated and coated fibres. For the uncoated fibres, the presence of the EOs, in particular BP-EOs, determined an increase of fibre ductility with respect to PLA fibres, as indicated by the higher values of elongation (Table 1 and Figure 4). These results are in line with the literature: previous studies have reported that the addition of lipid/oil components in polymer-based systems could weaken the interaction between polymeric chains, increasing the polymer chain mobility and, thus, leading to the formation of flexible regions within the polymer matrix [24,48,49]. The differences observed between PLA-BP and PLA-limonene could be due to the fact that BP-EOs, as already mentioned, contains not only limonene but also other chemical compounds that are less volatile than limonene and act as plasticisers for PLA [25].

The CS coating determined an increase in fibre mechanical resistance, as evidenced by the higher values of tensile strength measured for all coated fibres with respect to uncoated ones (Table 1). Only in the case of PLA fibres, the CS coating improved the tensile strength at the expense of ductility, making the fibres more brittle (Figure 4). The chitosan coating created a reinforcement layer around the fibres that increased fibre mechanical resistance but hindered their capability to elongate. As regards cPLA-BP and cPLA-L fibres, the CS coating did not significantly affect their ductility; this result could be explained by the presence of EOs working as plasticizers. The CS coating impacted on the toughness of the electrospun mats too, as shown in Table 1. An increase in toughness, which is the energy dissipated up to the point of break, was observed for all coated fibres.

The presence of the CS coating was further assessed by evaluating the wetting properties of the fibres, before and after CS treatment, by water contact angle measurements. In Table 2, the average values of the contact angle at T0 (0 min) and T5 (5 min) for the uncoated and coated PLA, PLA-BP and PLA-L fibres are compared. For the uncoated fibres, the water contact angle values remained constant over time (>120°) and values higher than 130° (enhanced hydrophobicity) were recorded for fibres containing BP and L due to the hydrophobic nature of the essential oils used. Contrary, the CS-coated fibres were more hydrophilic: at T0, contact angle values lower than 120° were recorded for all types of fibres indicating that the CS coating favoured the wetting of the fibrous membranes. This was even more evident at T5, when the water drop further spread onto the surface of the electrospun mat and the contact angle values tended to zero.

**Figure 5:**
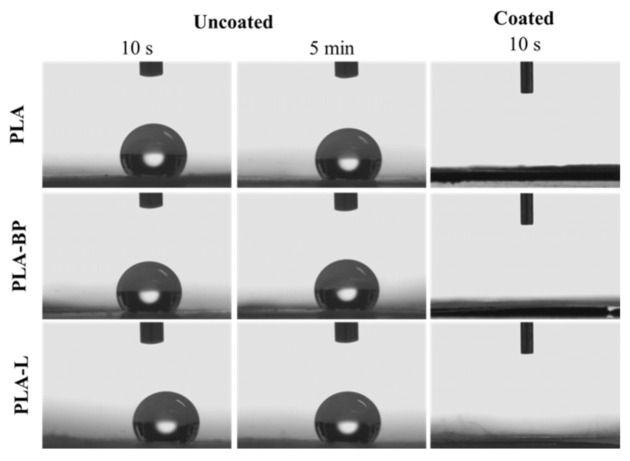
Images of a water drop on uncoated and coated PLA, PLA-BP and PLA-L fibres after 10 s and 5 min from drop dispensation.

Figure 5 shows the images of 5 µL water drops depostied on the surface of uncoated and coated fibres. It can be observed that, at the time points analysed (10 s and 5 min after release), the drop remains unaltered on the surface of the uncoated fibres, indicating the hydrophobicity of the fibres under investigation. Otherwise, it can be observed that, after 10 s, the drop is completely spread on the coated fibre surface, confirming that the coating determines a significant increase in fibrous membrane wettability and hydrophilicity.

**Figure 6:**
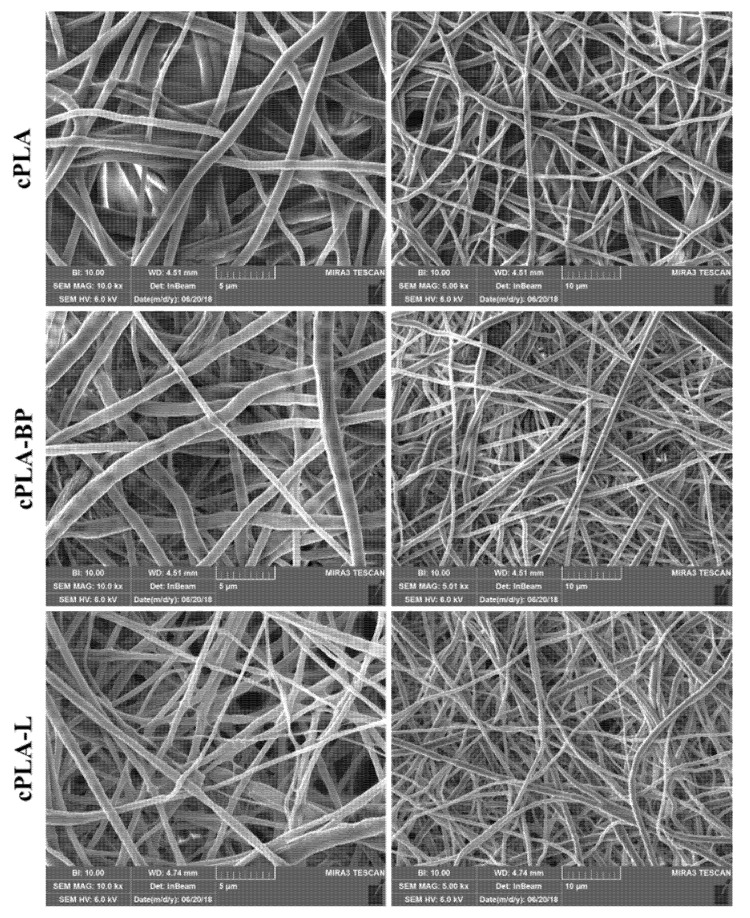
SEM micrographs, at different magnifications, of cPLA, cPLA-BP and cPLA-L fibres after immersion in water for 7 days. Scale bars: 5 µm (left hand side), 10 µm (right hand side).

The stability of the CS-coated fibres was assessed after soaking them in deionized water for 7 days (Figure 6). The morphology of the fibres remained almost unchanged, pointing out the stability of the fibrous membranes in aqueous solutions. In particular, the nano-pores on the surface of cPLA-BP and cPLA-L fibres were still closed, indicating that the CS coating persisted on the fibres’ surface.

**Figure 7:**
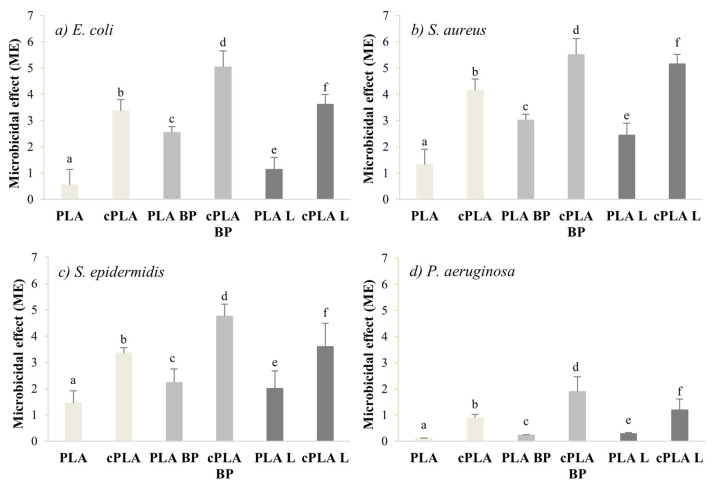
Microbicide effect (ME) of coated and uncoated fibres after 24h incubation with *E. coli* (**a**), *S. Aureus* (**b**), *S. Epidermidis* (**c**) and *P. Aeruginosa* (**d**) (mean values ± s.d.; n = 3). (**a**) ANOVA one-way (*p* < 0.05), Multiple Range Test: a vs. b, c; b vs. d; c vs. d, e; d vs. f; e vs. f. (**b**) ANOVA one-way (*p* < 0.05), Multiple Range Test: a vs. b, c; b vs. d; c vs. d; e vs. f. (**c**) ANOVA one-way (*p* < 0.05), Multiple Range Test: a vs. b; b vs. d; c vs. d; e vs. f. (**d**) ANOVA one-way (*p* < 0.05), Multiple Range Test: a vs. b; b vs. d; c vs. d; e vs. f.

The microbicide effect (ME) of both coated and uncoated fibres was evaluated on four different bacterial strains (*S. aureus*, *S. epidermidis*, *E. coli* and *P. aeruginosa*). Black pepper essential oil is known to be effective against *S. aureus* and *E. coli* at a Minimum Inhibitory Concentration (MIC) of 1–2 mg/mL [50,51,52], while MIC values in the range of 4–8 mg/mL have been reported for limonene against *S. aureus*, *E. coli* and *P. aeruginosa* [53]. It is therefore expected that the addition of the EOs imparts antibacterial properties to the PLA fibres. Indeed, the ME values recorded for PLA-BP fibres and PLA-limonene ones are considerably higher than the ME of pure PLA samples. The CS coating worked synergistically with the EOs and further enhanced the antibacterial properties of the electrospun membranes (Figure 7), due to the bactericidal effect of chitosan on gram-positive and gram-negative bacteria [54,55]. For all the microorganisms considered, the CS-coated fibres were characterized by ME values significantly higher than those of coated fibres. Among the coated fibres, cPLA-BP mats showed the highest antibacterial activity against all the microorganisms considered, pointing out the microbiocidal effect of the CS coating.

**Figure 8:**
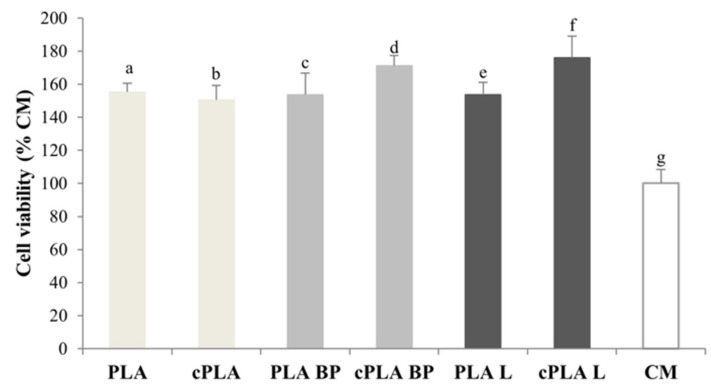
Cell viability (% CM) after incubation for 3 days with PLA, PLA-BP, and PLA-L fibrous membranes, coated and uncoated (mean values ± s.d.; n = 5). CM was considered as control. ANOVA one-way (*p* < 0.05), Multiple Range Test: a vs. g; b vs. g; c vs. g; d vs. e, f, g; e vs. g; f vs. g.

All types of fibres were tested on fibroblasts to evaluate their biocompatibility. Figure 8 reports the viability calculated for cells grown on PLA, PLA-BP and PLA-L fibres, uncoated and CS-coated, after incubation for 3 days. It can be observed that all the fibrous membranes are characterized by higher viability values with respect to culture medium (CM), indicating their complete biocompatibility. Moreover, a cell viability higher than 100% indicates that cells are able to penetrate the fibrous membrane and colonise it.

**Figure 9:**
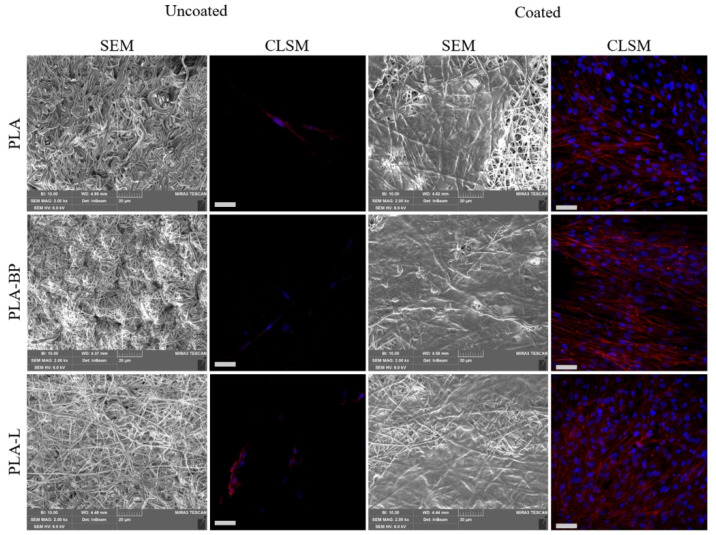
Microphotographs of fibroblasts grown on PLA, PLA-BP, PLA-L fibres, uncoated and coated, for 7 days: SEM images and confocal-laser scanning microscopy (CLSM) (nuclei in blue, Hoechst 33258; cytoskeleton in red, TRICT-phalloidin). Scale bars: 20 µm for SEM images, 50 µm for CLSM images.

Figure 9 shows SEM and CLSM microphotographs of fibroblasts grown on both uncoated and coated fibres after 7 days. It is evident that cell proliferation on uncoated fibres was limited, probably due to the high fibre hydrophobicity that limited cell adhesion. In contrast, SEM images of fibroblasts grown on cPLA, cPLA-BP and cPLA-L fibres reveal that the cells proliferated and grown on and inside the fibrous membranes, resulting in a complete colonisation. The adhesion and the proliferation of fibroblasts after 7 days of culture were also evaluated by CLSM analysis. It is evident that CS coating promoted cell adhesion and growth on the fibrous membranes, suggesting an optimal cells-substrate interaction. On the contrary, scaffolds without CS coating showed a poor interaction with the cells, as indicated by the total absence of cells on the scaffold surface.

## 4. Conclusions

Nano-textured fibres were electrospun from PLA solutions containing either black pepper EO or limonene. The poor miscibility of the EOs with PLA and the evaporation of the most volatile components of EOs induced phase separation events during electrospinning that led to the formation of nano-pores on the surface of the nanofibres. Such porous nanofibres were coated with a thin layer of chitosan to enhance their antibacterial properties, biocompatibility and capability to support cell adhesion and proliferation.

Water contact angle measurements showed that the presence of the chitosan coating made the PLA-based fibres hydrophilic, as also indicated by the almost instantaneous spreading of the water drops once in contact with the surface of the coated fibres. In addition, the mechanical properties of the composite systems were enhanced in terms of increased tensile strength and toughness. The chitosan coating worked as a reinforcement while maintaining or even improving the flexibility of the electrospun membranes (containing EOs). Antibacterial tests, performed on *S. aureus*, *S. epidermidis*, *E. coli* and *P. aeruginosa*, proved the synergistic effect of chitosan and EOs. Fibres containing BP-EO and limonene were characterised by a higher antibacterial activity with respect to those of pure PLA. All coated fibres exhibited a significantly higher microbicidal effect if compared to the uncoated fibres, pointing out that the presence of the chitosan layer positively impacted on the ability of the PLA-EOs fibres to stop bacterial growth.

In-vitro tests on fibroblasts revealed that all the fibrous membranes were biocompatible since they were characterized by cell viability values higher than that one of complete medium (control). Morphological and fluorescence imaging evidenced that the chitosan coating promoted cellular adhesion and proliferation. Indeed, only chitosan-coated fibres resulted completely colonized by cells.

This work demonstrates that the combination of essential oils and chitosan is beneficial to enhance the properties of PLA nanofibres (morphology, mechanical resistance, wetting properties, antibacterial activity, and biocompatibility) and develop composite fibrous membranes with potential applications in wound care.

## Figures and Tables

**Table 1:** Mechanical properties of uncoated and coated fibres (mean value ± s.e.; n = 3). ANOVA one-way (*p* < 0.05), Multiple Range Test: a vs. b, c, d; b vs. c, e; c vs. f; d vs. e, f; e vs. f; a’ vs. b’, d’; b’ vs. c’, e’; d’ vs. e’, f’; e’ vs. f’; a” vs. b”, c”, d”; b” vs. c”, e”; c” vs. f”; d” vs. e”, f”; e” vs. f”.

Fibres	Tensile Strength (MPa)	Elongation	Toughness (MPa)
PLA	0.61 ± 0.05 a	0.64 ± 0.02 a’	0.53 ± 0.04 a”
PLA-BP	0.91 ± 0.05 b	0.93 ± 0.26 b’	1.28 ± 0.09 b”
PLA-L	1.82 ± 0.18 c	0.64 ± 0.04 c’	1.65 ± 0.11 c”
cPLA	3.01 ± 0.74 d	0.44 ± 0.03 d’	1.29 ± 0.23 d”
cPLA-BP	1.32 ± 0.11 e	1.38 ± 0.13 e’	2.58 ± 0.94 e”
cPLA-L	2.75 ± 0.16 f	0.67 ± 0.06 f’	2.15 ± 0.35 f”

**Table 2:** Mean values of the contact angle at T0 and T5 for PLA, PLA-BP and PLA-L fibres, before and after the CS coating (mean values ± s.d.; n = 3).

Fibres	Water Contact Angle (°)	
	T0	T5 (min)
PLA	123 ± 3	121 ± 3
PLA-BP	133 ± 2	132 ± 1
PLA-L	131 ± 1	128 ± 2
cPLA	42 ± 3	n/a
cPLA-BP	116 ± 2	n/a
cPLA-L	118 ± 1	n/a

## Data Availability

Not applicable.
